# Traditional Bulgarian Dairy Products: Ethnic Foods with Health Benefits

**DOI:** 10.3390/microorganisms9030480

**Published:** 2021-02-25

**Authors:** Penka Petrova, Ivan Ivanov, Lidia Tsigoriyna, Nadezhda Valcheva, Evgenia Vasileva, Tsvetomila Parvanova-Mancheva, Alexander Arsov, Kaloyan Petrov

**Affiliations:** 1 Institute of Microbiology, Bulgarian Academy of Sciences, 1113 Sofia, Bulgaria; ivanov.ivnt@gmail.com (I.I.); alexander_arsov@abv.bg (A.A.); 2 Institute of Chemical Engineering, Bulgarian Academy of Sciences, 1113 Sofia, Bulgaria; lidinka29@gmail.com (L.T.); nadejda.130597@abv.bg (N.V.); jenivasileva96@gmail.com (E.V.); mila_parvanova@abv.bg (T.P.-M.)

**Keywords:** yoghurt, white brined cheese, *kashkaval*, *katak*, *kefir*, *koumiss*, LAB, probiotics, bioactive peptides

## Abstract

The reported health effects of fermented dairy foods, which are traditionally manufactured in Bulgaria, are connected with their microbial biodiversity. The screening and development of probiotic starters for dairy products with unique properties are based exclusively on the isolation and characterization of lactic acid bacterial (LAB) strains. This study aims to systematically describe the LAB microbial content of artisanal products such as Bulgarian-type yoghurt, white brined cheese, *kashkaval*, *koumiss*, *kefir*, *katak*, and the Rhodope’s *brano mliako*. The original technologies for their preparation preserve the valuable microbial content and improve their nutritional and probiotic qualities. This review emphasises the features of LAB starters and the autochthonous microflora, the biochemistry of dairy food production, and the approaches for achieving the fortification of the foods with prebiotics, bioactive peptides (ACE2-inhibitors, bacteriocins, cyclic peptides with antimicrobial activity), immunomodulatory exopolysaccharides, and other metabolites (indol-3-propionic acid, free amino acids, antioxidants, prebiotics) with reported beneficial effects on human health. The link between the microbial content of dairy foods and the healthy human microbiome is highlighted.

## 1. Introduction

Dairy products are indispensable, consumed daily and one of the most desired foods by a large part of the world population. They are prepared at home (artisanal, homemade, boutique dairy goods), or produced industrially in tonnage quantities everywhere in the world. To obtain them, fermentation technologies are used with the participation of various lactic acid bacteria (LAB). Lactic acid fermentation improves the taste, enhances the digestibility of the milk, and offers the manufactures a wide variety of valuable products. Fermented milk is very useful for many reasons. The related products have a prolonged shelf life; they are safe because LAB act as preservatives and inhibit the development of pathogenic microflora, they are extremely suitable for the absorption of nutrients from milk, and they are beneficial due to the health impact of lactic acid bacteria on various body functions [1].

According to Rul (2017), traces of fermented milk products (milk lipids discovered on clay vessels) have been found as early as 8000 B.C. in Asia Minor and Eastern Europe, soon after the domestication of milk-producing animals (cows, sheep, and goats) [2]. Evidence of *kefir* consumption was found in China in a Bronze Age tomb [3]. The first dairy products resembling yoghurt were invented around 5000–6000 B.C. in Mesopotamia [1]. Located on the Balkan Peninsula, between Western Europe and the Middle East, at this time the Bulgarian lands were inhabited by various ancient communities, with their typical material culture including fermented food traditions. The climate in Bulgaria is especially suitable for animal dairy husbandry. Bone fragments, collected from Azmashka Neolithic village (near Stara Zagora, 6000 B.C.), belonged to 118 cattle, 73 sheep, and 27 goats [4]. Historians believe that the Thracian tribe *Bizalti* (who inhabited today’s lands of Shumen, Targovishte and Varna) were the first to start purposefully preparing fermented dairy products [5]. Another direction in the search for the origin of lactic acid dairy fermentation is offered by the descriptions of the Greek historian Herodotus, according to whom the Scythians (nomadic tribes living between the rivers Dnieper and Don) consumed sour milk. Mare’s fermented milk was also used by the proto-Bulgarians for food, and stored in leather bags made of stomachs. The resulting product was called *koumiss* and was a staple food during military campaigns. The Slavs are known to have consumed *sura*, a product obtained by placing yoghurt in wooden barrels in the summer and consuming it in the winter by liquefying it with drinking water. When the ancient Bulgarians rediscovered sheep’s yoghurt used by the Thracians and Slavs, it became preferable to mare’s milk. The observation that fermented dairy products are beneficial for human health dates back to their invention since it is described in Indian Ayurvedic scripts from about 6000 B.C. [6]. In Europe, the healing effects of Bulgarian yoghurt have been known since at least 1542, when the French King Francois I was cured of chronic diarrhoea by a simple yoghurt diet [2]. However, the discovery of yoghurt microbiota (as a cause of yoghurt fermentation) happened only in the 20th century. In 1905, Stamen Grigorov, a Bulgarian medical student in Geneva, Switzerland, was the first to describe the rod-shaped lactic acid bacterium (named *Bacillus bulgaricus* Grigoroff), accompanied by a spherical *Streptococcus*, in Bulgarian yoghurt [7]. Based on Grigorov’s findings, in 1909 the Russian biologist and Nobel Prize winner Elie Metchnikoff, developing his theory about the prolongation of life, was the first who proposed that daily yoghurt consumption engenders the longevity of the Bulgarian peasant population, especially in the mountainous regions. Metchnikoff suggested that there is a connection between the consumption of yoghurt and the number of Bulgarian centenarians. He further proposed the hypothesis that the inhibition of harmful food fermentation in the gut can delay the process of ageing. At the heart of his research is lactic acid, which reduces the number of putrefactive microorganisms [7,8]. Then, the benefits of yoghurt consumption were widespread in Europe by doctors, pharmacologists and journalists, which led to a general demand for yoghurt as a medicine in the first third of the 20th century, for instance, against “food neophobia” and other gastrointestinal disorders. In the period 1909–1912, physicians and bacteriologists Guéguen, Bulloch, Vaughan, Hertz, and Lane independently of each other discussed the therapeutic nature of the “lactic acid bacillus”, and as a result, Danone’s company distributed its yoghurt through the city pharmacies of Barcelona in 1912, followed by entering the French yoghurt market in 1923 [7]. Today, more than 45 bln metric tons (MT) of fresh dairy products are consumed annually in Europe. In 2019, 6.4 bn MT of cheese and 6.1 bn MT of yoghurt were produced by the countries of the European Union. Bulgarian dairies processed 663,644,000 L of raw milk in 2020, 94.3% of which was cow’s milk. In comparison to the other EU countries, the obtained genuine yoghurt (156,610 MT) and white brined cheese (899 MT) in Bulgaria were in limited quantities, but they are known for their very high quality [9].

## 2. Overview of Traditional Bulgarian Dairy Products: Appearance, Nutritional Value, Production Technologies, and Shelf Life

### 2.1. Yoghurt

Bulgarian yoghurt is a traditional Bulgarian dairy food, a hallmark of the country, produced by microbial lactic acid fermentation of pasteurized milk, inoculated with a starter culture of only two lactic acid bacterial species—*Lactobacillus delbrueckii* subsp. *bulgaricus* (*L. bulgaricus*) and *Streptococcus thermophilus*. Usually, Bulgarian yoghurt is prepared from cow’s milk, but it could also be produced from buffalo’s, goat’s or sheep’s milk, and according to the FAO/WHO definition (1984), it should contain at least 10^7^ viable colony-forming units (CFU) of symbiotic starters per gram product [10]. According to EU hygiene regulations, Bulgarian yoghurt industrially produced from raw milk could contain a maximum of 10^6^ bacterial (CFU) and up to 10^5^ somatic cell count (SCC) in mL of cow’s milk [11]. Another important requirement for raw milk is the absence of inhibiting substances (antibiotics, or natural plant compounds), which could inhibit the growth of the LAB starter culture. The total acidity of raw milk should not exceed 18–23 °T (Table 1).

Bulgarian yoghurt is white, sometimes slightly yellowish. Its surface could have a visible fat layer. The coagulum of the product is dense, slick and smooth. When tilted, the coagulum could rupture and exude some clear, slightly yellowish and opalescent milk serum. When buffalo’s or sheep’s raw milk is used, yoghurt consistency could be either homogeneous and creamy, or granular. The taste and aroma are specific, pleasantly sour and depend on the type of milk. According to the International Dairy Federation (IDF) Standard 99A (1987) Sensory Evaluation of Dairy Product of the International Dairy Federation in Brussels, yoghurt is evaluated by five indexes: appearance, colour, taste, odour and consistency [12].

Both artisanal and industrial technologies for yoghurt production are described as follows. They start with raw milk filtration to remove solid particles, homogenization and pasteurization by heating to 93–95 °C for 3–5 s and cooling to 45–50 °C. Then, the raw milk is inoculated with a starter culture, consisting of *L. bulgaricus* and *S. thermophilus*, in the ratio between 1:2 and 1:5. The starter culture is 0.5 to 2% of the raw milk. The inoculated, still liquid and warm milk is spilt in sterile vessels or packages, usually from 150 g to 1 kg. The packages are capped immediately after filling and incubated in a warm place or thermal chamber at 42–45 °C for 2.5–4.0 h. LAB quickly multiply and reach 10^7^–10^8^ CFU/g (*L. bulgaricus*) and 10^8^–10^9^ CFU/g (*S. thermophilus*). LAB ferment the lactose to lactic acid, which binds calcium ions (Ca^2+^) from casein in milk, thus obtaining its coagulation. For the production of high-quality yoghurt, the temperature during fermentation should be stable, and there should not be vibrations. The final product is cooled to 6–12 °C and could be stored for up to 21 days.

Yoghurt is considered to be a nutrient-dense food that significantly contributes to the intake of macro-and micro-nutrients, most importantly calcium and protein. It is a source of iodine, potassium, phosphorus and vitamins: the water-soluble riboflavin (B2) and cobalamin (B12), and fat-soluble secosteroids such as vitamin D.

### 2.2. White Brined Cheese

**Figure 1:**
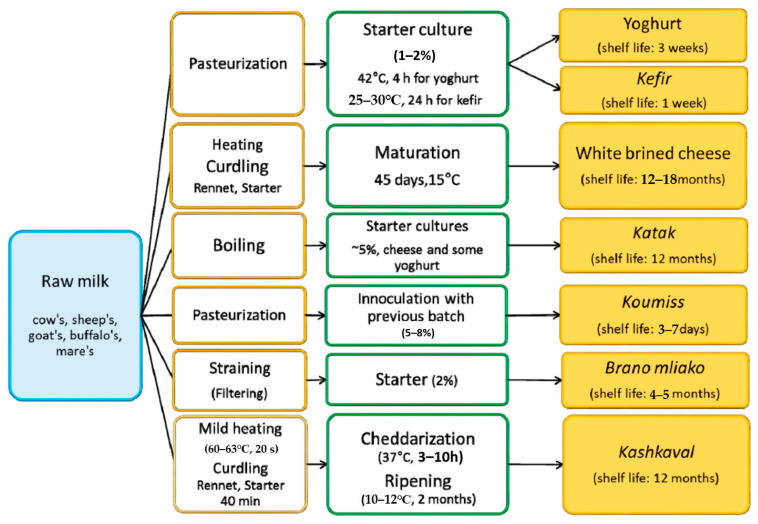
Technological steps of preparation of traditional Bulgarian dairy products.

White brined cheese is the second most widespread and consumed traditional dairy product after yoghurt. It originated from Bulgaria and was widely distributed during the second half of the 20th century in the countries of the Balkan Peninsula, the former Soviet republics, Turkey, the Middle East, and Mexico. It is prepared from cow’s, sheep’s, goat’s, buffalo’s or mixed milk, which is fermented and subsequently stored in brine. White brined cheese is obtained by hardening the milk with a special technology (Figure 1).

Cheese that is made from two different types of milk usually has improved nutritional characteristics [13]. White brined cheese belongs to the group of hard cheeses with low water and high solids content, requiring a long ripening process of intensive acidification, proteolysis, and lipolysis. Its quality is determined by chemical, physical, microbiological, and organoleptic characteristics which guarantee safety, prolonged shelf life and high nutritional value of the product. One of them, for assessing the appearance of the cheese, is the “cut surface”. According to the National Bulgarian Standard, the cheese lump must appear as a cube with rectangular walls and a homogeneous, porcelain scrap cut surface, without the presence of bacterial pores and layering [14].

In general, the process first involves preheated milk, which is cooled and fermented using rennet, the enzyme mixture that curdles the casein in milk. Rennet contains the aspartic endopeptidase chymosin, pepsin, and lipase [15], and is usually isolated from the abomasum of new-born small ruminants, but also alternatively manufactured by fermentation using recombinant *E. coli*, *Aspergillus niger* var. *awamori*, or *Kluyveromyces lactis* [16]. The next technological steps are the straining of the coagulate, cheese maturation for a minimum of 45 days at 15°C, salting and packaging in boxes containing brine (6–10% Sodium chloride, Thörner degrees of acidity 160–180 °T). The shelf life of the product is 12–18 months.

When prepared from cow’s milk, the protein content in white brined cheese is 80% αS1, αS2, β and κ-casein, and 20% whey proteins—α-lactalbumin and β-lactoglobulin. The physical properties and chemical content of Bulgarian white brined cheese are presented in Table 2.

White brined cheese has high energy and nutritional value, which is due to the proteins and fats, but also the presence of easily digestible peptides, essential free amino acids (leucine, isoleucine, lysine, methionine, cystine, phenylalanine, tyrosine, tryptophan, and valine), vitamins (A, B, E, and D), minerals (calcium, zinc, magnesium, and phosphorus), and lactic acid [17,18]. Its unique taste and aroma are due to the rich spectrum of volatile components, such as aldehydes, alcohols, carboxylic acids, methyl ketones, ethyl esters, sulphur compounds, and aromatic hydrocarbons [19].

### 2.3. Kashkaval (Yellow Cheese)

*Kashkaval* also belongs to the hard cheeses and is the typical yellow cheese in Bulgaria. Yellow cheese is a dairy product produced mainly on the Balkan Peninsula and in the Mediterranean. According to National Bulgarian Standard BDS 14:2010, *kashkaval* is produced from different kinds of milk (Table 3). When it is made from sheep’s milk, it is called “Balkan”, the one from cow’s milk is called “Vitosha”, and the one from a mixture of the two kinds of milk is called “Preslav”. However, just like cheddar cheese, the homemade *kashkaval* could also be made, albeit rarely, from goat’s milk. Common to all technologies is the mandatory process of cheddarization. It takes a certain period (2 to 6 months) to develop its specific and characteristic aroma.

According to Aladjadjiyan et al. (2016), homemade *kashkaval* is made from milk with at least 3.5% fat content, without any additional ingredients [20]. Briefly, the technology includes the pre-filtering and centrifugation of the raw milk, and heating at 60–63 °C for 20 s to reduce the number of spoilage microflora, and at the same time, to preserve the beneficial LAB. The milk is then cooled and poured into a bath. Rennet is added and curdling occurs in 30–40 min. The coagulum is cut into small pieces with a size of 0.5 cm, and whey is released. After some time, the whole mass is stirred for about 20 min, then heated to 38–39 °C with continuous stirring. The procedure is called “cheese baking”. By artisanal recipes, cheese can also be “boiled” at this stage by the addition of weak acids, such as citrate or acetate. Upon completion, the grains become firm and elastic, and the whey is drained. A cheddarization process follows. It lasts 2–3 h at a temperature of 35–37 °C and is due to the rapid lactic acid fermentation which causes: (*i*) the multiplication of LAB that break down lactose to lactic acid, (*ii*) lowering the pH to 5.4–5.2, and (*iii*) calcium extraction from calcium paracaseinate to obtain monocalcium paracaseinate, a soft and malleable material allowing easy compression and formation of the final product. Thus, *kashkaval* acquires plastic properties and is considered ready when it emits a tympanic sound on being struck. The cheese is then cut into thin slices and placed in a concentrated salt solution, which helps to remove water and increases the firmness. Finally, it is boiled for a few minutes, and the resulting slurry is kneaded and placed in moulds. After one day, it is separated from the mould and left to mature at about 8–12 °C for ~55 days. After ripening, *kashkaval* can be covered with paraffin or dipped in melted wax to prevent it from drying out. The lactic acid bacteria in the starter ultimately determine the taste of *kashkaval* during its ripening, which is a slow and expensive process requiring special chambers allowing temperature control and appropriate humidity [21]. The data in Table 4 show that during 12 months of storage, at four different temperature regimes (4.0 °C, 1.0 °C, −7.5 °C and −18.0 °C), the main physicochemical parameters of *kashkaval* from cow’s milk, namely water content, dry matter, total protein, total fat content and salt content, do not change significantly. The ratio of water-soluble to total nitrogen is significantly influenced by the storage temperature. A higher temperature allows longer glycolysis and proteolysis in *kashkaval* (Table 4). 

The data concerning viable bacterial content of the product indicated greater sensitivity to the impact of low temperatures on *Lactobacillus* spp. compared to *Streptococcus* spp. Markova et al. (2019) reported a reduction in the number of viable lactobacilli by one logarithm after storage for 12 months in a refrigerated (−7 °C) and frozen (−18 °C) state, while the number of viable cells of *Streptococcus* spp. remained unchanged.

### 2.4. Katak

*Katak* or *krotmach* (*kurtmach*) is a specific Bulgarian salty-tasting dairy product. The name is used for several products with a similar taste but produced by different recipes. Traditionally, *katak* is obtained by inoculating sheep’s milk with cheese used as a starter culture. The resulting product is durable and can be stored for several months [20,24]. *Katak* can be prepared from boiled or non-boiled milk, but the first is considerably tastier. To obtain a thicker and denser product, sheep’s milk should be boiled over low heat for several hours (or days) before inoculation. Ancient technology was described in an ethnographic study of Aladjadjiyan et al. (2016) as follows: the starting material should be sheep’s milk collected in August when milk is relatively dense, and it is further thickened by boiling in a water bath (in a small container and immersed in a larger water tank). The milk is boiled continuously and stirred occasionally with a wooden spoon for several hours until the desired creamy consistency is obtained. After cooling, white brined cheese (as a starter culture) and some salt are added. In the past, it was usually stored in well-washed sheepskins; the residual fluid is released through the pores of the skin and this makes the *katak* tastier. Then, it may be stored in glass or plastic jars in a cool place. Nowadays, *katak* is often a homogeneous mixture composed of yoghurt and sheep’s cheese, to which butter may also be added. However, this is not an authentic product. Due to the salt content, organoleptically *katak* has a mixed taste between yoghurt and soft-ripened cheese. It contains lower fat (10%) than cheese and a low level (1%) of carbohydrates [25]. Its shelf life is more than 12 months.

### 2.5. Kefir

*Kefir* originated from the Caucasian mountains, Russia, and Tibet, and later became popular in Central and Eastern Europe. Its production is due to the combination of lactic acid and alcoholic fermentation. Since the final product is a thick drink containing carbon dioxide, *kefir* belongs to the carbonated dairy beverages giving a “sparkling” sensation, in addition to the specific tart taste and slightly alcoholic aroma [26].

In Bulgaria, *kefir* could be either natural (plain) or with added fruits (flavoured). Fermentation is initiated by the addition of kefir grains to raw milk. The original kefir grains are white to slightly yellowish, 0.3 cm in diameter, cauliflower-shaped, and semi-hard granules, containing polysaccharides, fats, proteins, and the symbiotic association of yeasts and bacterial strains [26,27,28]. The grains are insoluble in water, but when suspended in milk, they swell and form a gelatin-like product, the polysaccharide kefiran consisting of repeated hexasaccharide units [26]. Kefiran is a hetero-polysaccharide with glucose and galactose units. LAB are the main exopolysaccharide producing microorganisms and are responsible for the texture and the rheological properties of *kefir* [29].

The technology of *kefir* preparation allows the use of sheep’s, cow’s, or goat’s milk. For large-scale production, kefir grains are not used, but rather sterilized milk is incubated directly with selected microorganisms. On an industrial scale, the milk is sterilized by pasteurization after homogenization, kept for cooling down to 20 °C, inoculated and incubated with specific strains for 24 h [30,31]. Total acidity in the range 95–100 °T (pH 4.0–4.5) indicates the completion of the fermentation process and the formation of a stable coagulate [32].

### 2.6. Koumiss

*Koumiss* is a traditional milk beverage in Central Asia, China, Russia, and Kazakhstan, and, named *airag*, is the national drink of Mongolia [33]. The technology for its production was most probably transferred to Europe by Bulgarians around the 7th century. *Koumiss* is usually produced through the fermentation of raw milk by indigenous LAB and yeast [34,35]. Similar to the *kefir*, it is a slightly alcoholic, lactic acid-rich beverage (Table 5), but it is prepared by a liquid starter of the previous day batch inoculated to fresh milk and kept for about 8 h of fermentation. However, differing from *kefir*, which could be made from all known kinds of milk, *koumiss* is prepared only from mare’s and camel’s milk, which are high in sugar (6.3% lactose) and low in fat (12.1%). Nowadays, the large-scale production of *koumiss* is performed from cow’s milk supplemented with sugar to reach the composition of mare’s milk [36]. After fermentation, *koumiss* generally contains about 2% alcohol, 0.5–1.5% lactic acid, 2–4% sugars and 2% fat [37]. Depending on the lactic acid and ethanol content, *koumiss* is categorized into mild, medium and strong [34].

### 2.7. Rhodope’s Brano Mliako

Almost forgotten today, the so-called *brano mliako* has been a traditional dairy product in the Rhodope Mountains since ancient times. Known for its qualities as one of the most unique organic foods in the world, this original Rhodope dairy product is prepared according to an ancient recipe that has remained unchanged for centuries. *Brano mliako* resembles yoghurt, but is made of ewe’s milk at the end of summer only. The raw milk is collected for days in special wooden containers, where it is filtered and thickened by significant dehydration. The thick milk ferments spontaneously, or is supplemented with some starter culture (sheep’s yoghurt), and is then “sealed” with a thin layer of sheep or goat tallow. Thus, reliably preserved, the *brano mliako* is suitable for use in the next three or four months, with fully preserved taste. A laboratory method, developed by Gruev (1970), obtained the same product and described the technology. This includes a two-fold concentration of milk at reduced pressure (45–50 °C), sterilization by the Koch method for 30 min; cooling to 45 °C, the addition of 1% yoghurt starter, fermentation to ~190 °T acidity; the addition of 2% yeast starter, isolated from *brano mliako* (cultured in grape must), fermentation; filling into glass containers at the end of yeast fermentation, hermetic sealing, and storage at 8–10 °C for at least four months [38]. Whether produced by traditional or laboratory methods, *brano mliako* is popular among scientists who are looking for the reasons behind the longevity of the peasant population in the Rhodope Mountains.

## 3. Microbial Content of Traditional Bulgarian Dairy Products

**Figure 2:**
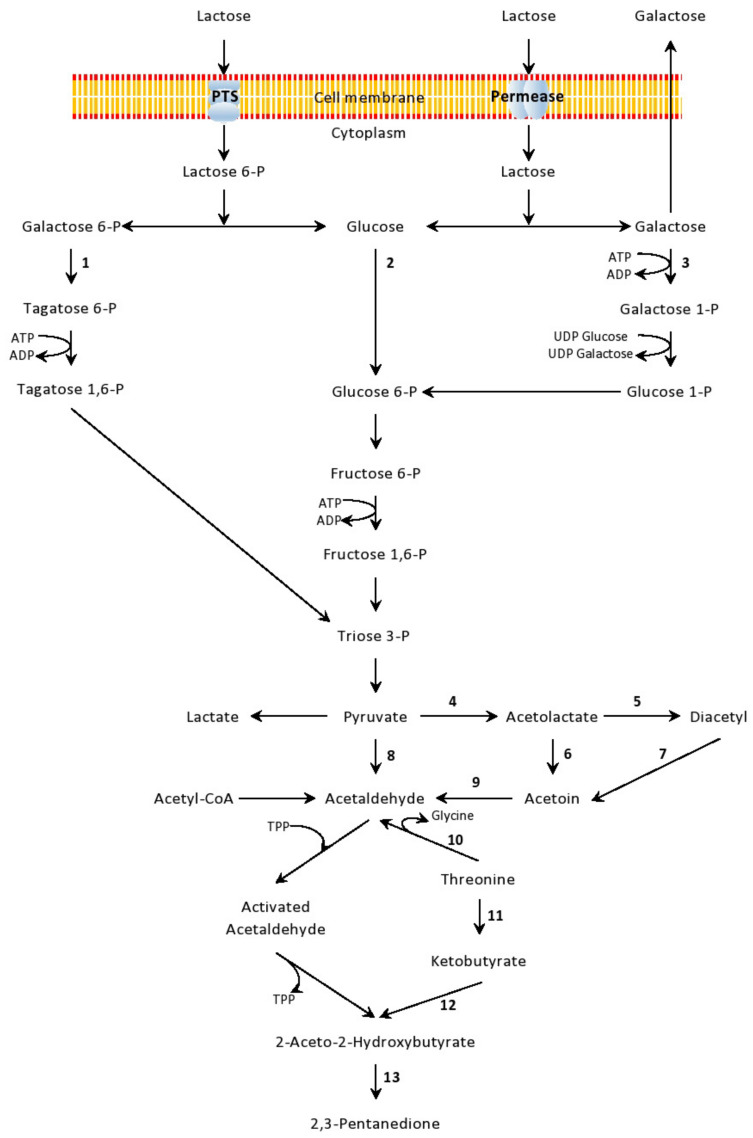
Schematic presentation of lactose transport, metabolization routes and formation of volatile compounds by lactic acid bacteria (LAB) in dairy foods, according to Rul (2017), and Solopova et al. (2012) [2,42]. Designations: 1, tagatose 6-phosphate pathway; 2, glycolysis; 3, Leloir pathway; 4, acetolactate synthase; 5, oxidative decarboxylation; 6, acetolactate decarboxylase; 7, diacetyl reductase; 8, pyruvate decarboxylase; 9, acetoin dehydrogenase; 10, threonine aldolase; 11, threonine deaminase; 12, acetolactate synthase; 13, oxidative decarboxylation.

Genuine Bulgarian yoghurt contains a starter culture consisting of L. delbrueckii ssp. bulgaricus and S. thermophilus. Their proto-cooperation was comprehensively reviewed in several studies [10,39,40,41]. Briefly, L. bulgaricus, which is a bacterial species with high proteolytic activity, releases free amino acids that favour the growth of S. thermophilus. On its own, S. thermophilus engenders rapid acidification and diminishes the levels of dissolved oxygen in milk, thus improving the culture conditions for its symbiotic partner. Lactose is the main carbon source for LAB growth in milk, and different species have developed various mechanisms to transport and utilize it. Figure 2 shows the routes of lactose conversion to lactic acid and other volatile compounds contributing to the unique flavour of dairy foods.

Two forms of lactose can be introduced into the cell, unphosphorylated or phosphorylated. The first engages the transporter lactose permease LacS. Inside the cell, the enzyme β-galactosidase hydrolyses lactose to glucose and galactose [43]. Depending on the LAB species, lactose transport is coupled to proton symport or galactose antiport [2]. *L. bulgaricus* and *S. thermophilus* release the galactose moiety of lactose into the medium, whereas *Leuc. lactis*, *Lactococcus* ssp., and many *Lactobacillus* ssp. metabolize it. Glucose is metabolized via glycolysis, whereas galactose, depending on the particular LAB, follows either the tagatose 6-phosphate or the Leloir pathway [44,45]. Other LAB, such as starter lactococci, *L. paracasei*, *L. casei*, and *L. plantarum*, import lactose exclusively by *lac*-PTS—the phosphoenolpyruvate (PEP)-dependent phosphotransferase system. In this way, lactose is translocated and phosphorylated simultaneously with PEP as the first phosphoryl donor. After translocation, lactose is hydrolysed to glucose and galactose-6-P; glucose enters the glycolytic pathway through phosphorylation by glucokinase, whereas galactose-6-P is further metabolized via the tagatose-6-P pathway. However, pyruvate, aspartate and aromatic amino acids are the key metabolites needed for the production of the wide spectrum of volatile compounds contributing to the specific aroma of dairy foods [46,47]. As for Bulgarian yoghurt, its sour smell is associated with high amounts of lactic acid, but the overall flavour is also influenced by acetaldehyde, acetoin, diacetyl, and 2,3-pentanedione [48].

When artisanal yoghurt is prepared, it contains rich autochthonous LAB microflora. According to Velikova et al. (2018), 53% of the LAB strains isolated from homemade yoghurts belong to *L. bulgaricus*, 14% to other lactobacilli, and 32% to lactic acid cocci (*S. thermophilus*, *Pediococcus acidilactici*, *Lactococcus lactis*, *Enterococcus faecium*). In Table 6 are shown the most common lactobacilli in Bulgarian yoghurt, which are *L. helveticus*, *Lacticaseibacillus paracasei*, *Limosilactobacillus fermentum*, and *Lacticaseibacillus rhamnosus*; several strains of *Leuconostoc mesenteroides*, *Leuc. pseudomesenteroides*, and *Weissella confusa* were isolated from yoghurt as well [10].

Regarding white brined cheese, the starter culture usually contains *Lc. lactis* subsp. *lactis* and *Lacticaseibacillus casei*, *L. bulgaricus* and *S. thermophilus*. The majority of non-starter LAB are mesophilic lactobacilli such as *Lactiplantibacillus plantarum*, *L. paraplantarum*, *L. pentosus*, *L. paracasei* subsp. *paracasei*, *Lentilactobacillus hilgardii*, and *L. brevis* (Table 6). Most of them are salt- and acid-tolerant facultative anaerobes, which grow well in cheese as their number reaches up to 10^9^ CFU/g during ripening [49]. Other authors report an extremely large amount of LAB, up to 3.7 × 10^10^ CFU/g, observed in white brined cheese produced by a small family farm near Dryanovo, Bulgaria [50]. Four different strains of *L. plantarum*, *Pediococcus* spp., *Enterococcus* spp., and *Leuconostoc* spp. with probiotic properties have been isolated from Bulgarian home-made brined cheese [51]. Four bacteriocinogenic strains of *Ent. faecium* have been isolated and identified from Bulgarian home-made white brined cheese, and the authors propose to use them as preservatives in the production of dairy products [52]. The species *Ent. faecium* and *Ent. durans* are used as adjunct culture in the manufacture to accelerate the proteolysis of β-casein and αs1-casein [49].

*Kashkaval* microflora was recently described by Teneva-Angelova et al. (2018) [53]. The main starter cultures used in its production include the mesophilic *Lc. lactis* ssp. *lactis* and *Lc. lactis* ssp. *cremoris*, and the thermophilic *L. bulgaricus*, *L. helveticus*, and *S. thermophilus*. A broad spectrum of other species has been isolated from artisanal samples contributing to their flavour: *Leuc. mesenteroides*, *L. lactis* ssp. *lactis* biovar. *diacetylactis*, and *Enterococcus* spp. [54,55].

The study of Tserovska et al. (2002) showed that 18 different LAB strains can be isolated from home-made *katak*. Nine of them belonged to the lactic acid cocci (*P. acidilactici*, *P. pentosaceus*); the others—to *L. delbrueckii* ssp. *bulgaricus*, *L. delbrueckii* ssp. *delbrueckii*, and *L. delbrueckii* ssp. *lactis*) [25]. The predominance of lactic acid cocci in homemade cheese and *katak* was reported by Kirilov et al. (2011). From 110 identified LAB strains, 58 belonged to genus *Enterococcus*, 20 to *Streptococcus* spp., 11 to *Lactococcus* spp., and only 21 were identified as *Lactobacillus* spp. [56]. Typical for *kefir* are LAB species, *L. kefiranofaciens*, *Lentilactobacillus kefiri* [57], *Lentilactobacillus buchneri* [58], *L. plantarum* [57], and the yeast species *Kluyveromyces marxianus* [59], *Kazachstania unispora* [60], *Dekkera anomala* [61], and several species of the genus *Saccharomyces* [62]. *Koumiss* is fermented mainly by LAB species, *L. acidophilus*, *L. helveticus*, *Ligilactobacillus salivarius, L. buchneri*, *L. plantarum* [34], *S. thermophilus*, and *Leuconostoc* spp. [63], and yeasts *Torula kumiss*, *Saccharomyces lactis*, *Sacch. unisporus*, and *Kluyveromyces lactis* [35,36].

**Table 6:** The microbial content of traditional Bulgarian dairy foods and beverages.

Product	Starter Strains	Accompanying Microflora	Reference
Yoghurt	*L. delbrueckii* ssp. *bulgaricus, S. thermophilus*	*L. helveticus, L. paracasei, L. fermentum, Lacticaseibacillus rhamnosus, Leuc. mesenteroides, Leuc. pseudomesenteroides, W. confusa, P. acidilactici, Lc. lactis, Ent. faecium*	[10,39,40,41]
White brined cheese	*Lc. lactis* ssp. *lactis, L. casei, L. delbrueckii* ssp. *bulgaricus, S. thermophilus*	*L. plantarum, L. paraplantarum, L. pentosus, L. paracasei* ssp. *paracasei, Lentilactobacillus hilgardii, L. brevis,* *Leuconostoc* spp., *Ent. faecium, Ent. durans*	[49,50,51,52]
*Kashkaval*	*Lc. lactis* ssp. *lactis, Lc. lactis* ssp. *cremoris, L. delbrueckii* ssp. *bulgaricus, L. helveticus, S. thermophilus*	*Leuc. mesenteroides, L. lactis* ssp. *lactis* biovar. *diacetylactis, Enterococcus* spp.	[53,54,55]
*Katak*	*L. delbrueckii* ssp. *bulgaricus, S. thermophilus*	*L. delbrueckii* ssp. *delbrueckii*, *L. delbrueckii* ssp. *lactis, P. acidilactici, P. pentosaceus, Enterococcus* ssp.	[25,56]
*Kefir*	*L. kefiranofaciens, Lentilactobacillus kefiri, Lentilactobacillus buchneri, L. plantarum, L. amylovorus, Levilactobacillus brevis, L. casei, L. paracasei, L. crispatus, Lactobacillus delbrueckii subsp. bulgaricus, L. helveticus, L. parakefiri, L. satsumensis, L. uvarum, S. thermophilus, Lc. lactis* ssp. *cremoris, Lc. lactis* ssp. *lactis, Kluyveromyces marxianus, K. lactis*	*Leuc. lactis, Leuc. mesenteroides, Acetobacter fabarum, A. lovaniensis, A. syzygii, Ent. faecium, Gluconobacter japonicus, Weissella* spp., *Halococcus* spp., *Candida inconspicua, Dysgonomonas* spp., *Geotrichum candidum, Kazachstania aerobia, Kz. exigua, Kz. unispora, Lachancea meyersii, Pelomonas* spp., *Pichia fermentans, P. guilliermondii, P. kudriavzevii, Sacch. cerevisiae, Sacch. martiniae, Sacch. turicensis, Sacch. unisporus, Shewanella* spp.	[57,58,59,60,61]
*Koumiss*	*L. acidophilus, L. helveticus, Ligilactobacillus salivarius, S. thermophilus, K. lactis*	*L. buchneri, L. plantarum, Leuconostoc* spp., *Sacch. lactis, Sacch. unisporus, Torula kumiss*	[34,35,36,62]

## 4. Health-Promoting Metabolites in Traditional Bulgarian Dairy Products

Dairy products have been recognized as functional foods for decades because of the accumulated scientific evidence of positive effects on the overall health of the consumers. Among the most important health-promoting components of dairy products are bioactive peptides with various functions, fatty acids (γ-aminobutyric acid, conjugated linoleic acid), lactic acid and exopolysaccharides. All these compounds have immunomodulatory, antihypertensive, antitumor or anticancer activity, as well as antimicrobial, antioxidant and mineral-binding properties [53]. A large part of the world adult population is unable to digest lactose, which comprises 4–6% of the milk content. Yoghurt, cheese, *katak*, and *koumiss* are suitable dairy products for lactose-intolerant patients. *L. bulgaricus* can diminish more than two-fold the lactose content in yoghurt [10]; the same effect was reported for LAB in *kefir* and *koumiss* [34].

### 4.1. Free Amino Acids (FAA)

LAB are known for their abundant protease and proteinase enzymes, hydrolysing more than 40% of the peptide bonds of caseins (αs1-, αs2-, β-, and κ-casein) in milk, which results in the formation of free amino acids, dipeptides, and more than 100 different oligopeptides during fermentation [2]. *L. bulgaricus* strains are auxotrophic for 15 to 20 amino acids and can synthesize only aspartate, asparagine, threonine, and lysine [61]. However, this is the LAB species possessing higher overall levels of proteolytic activity, much higher than that of *S. thermophilus*, and it liberates most of the amino acids needed for the growth of its symbiotic partner, including valine, histidine, glycine, leucine, isoleucine, and methionine [39]. The process is initiated by a cell-envelope proteinase, which forms oligopeptides that are subsequently digested to shorter peptides by the concerted action of various intracellular peptidases [63].

The high proteolytic activity of LAB leads to increased levels of free amino acids (FAA) in dairy products. As part of the accompanying microflora of yoghurt, *L. fermentum*, *L. paracasei*, and *P. acidilactici* release high amounts of lysine and tryptophan, while *L. helveticus* accumulates L-arginine and its precursor L-citrulline in homemade Bulgarian yoghurts [41]. The last two amino acids are used in the treatment of cardiovascular diseases, irritable bowel syndrome, endothelial dysfunction, hypertension, heart failure, atherosclerosis, and ischemia-reperfusion injury [64]. Increased FAA content was also reported for cheese. On the 45th day of cheese ripening, it increases three-fold and reaches 154 mg per 100 g product [65]. *L. helveticus* isolated from *kefir* was also reported to reach 53.38 mg essential FAA per 100 g product, 1.5 times greater than the content in yoghurt [66].

### 4.2. Bioactive Peptides

Some of the peptides produced by LAB are known as phospho-peptides. They accelerate the mineral absorption of calcium, phosphorous, iron, and magnesium [67]. Different studies show a link between dairy intake and bone turnover markers and a positive association with bone mineral content. The favourable changes in biochemical indexes of bone metabolism are superior to those offered by calcium supplementation alone. Daily consumption of dairy products diminishes the risk of bone fractures [68].

Bacteriocins are known as bioactive peptides preventing dairy food spoilage and contributing to the overall gut health of the consumer. For example, *Ent. faecalis* and *Ent. faecium*, used as an adjunct culture for the production of white-brined cheese, secrete a set of bacteriocins: enterocin A, enterocin B, enterocin P, enterocin 50, bacteriocin 31, and AS-48 cytolysin, with a strong inhibitory effect on *Listeria monocytogenes, Staphylococcus aureus, Clostridium botulinum, C. perfringens,* and *Vibrio cholerae* [69]. Four novel strains of *L. plantarum* (RL29, RL34, RL36, and RL37) isolated from Bulgarian home-made white brined cheese have been designated as bacteriocin producers and evaluated as promising probiotics [51]. Another seven bacteriocin-producing LAB strains belonging to *L. rhamnosus, L. bulgaricus, Lc. lactis* ssp. *lactis, Ent. faecium, L. plantarum,* and *L. casei* have been isolated from authentic Bulgarian dairy products [70]. The most interesting among them is *L. bulgaricus* strain BB18, isolated from *kefir* and a potent producer of *bulgaricin* [71]. It possessed the highest activity and the widest antimicrobial spectrum against both Gram-positive and Gram-negative pathogenic bacteria, and importantly, against *Helicobacter pylori*. *L. brevis* isolated from *katak* was evaluated as a putative bacteriocin-producing candidate with antibacterial and antifungal (against *Aspergillus* and *Penicillium*) activity [71].

Two cyclic peptides, cyclo(phenylalanyl-prolyl) and cyclo(leucyloprolyl), with antimicrobial, antiviral, antiprotozoal, antiparasitic, and antitumor activities, as well as radioprotective effect, were recently reported in Bulgarian yoghurt. Both peptides were produced by *S. thermophilus* and LAB of the accompanying microflora (*L. fermentum*, *Leuc. mesenteroides*, and *P. acidilactici*) [41].

Another important class of bioactive peptides, found mainly in yoghurt and white brined cheese, are those with angiotensin-converting enzyme (ACE-I)-inhibitory activity. The consumption of products rich in these peptides results in an overall antihypertensive effect. Probiotic yoghurt containing a mixed starter of *L. delbrueckii* ssp. *bulgaricus* Lb1466, *S. thermophilus* St1342, *L. acidophilus* L10, *L. casei* L26, and *Bifidobacterium lactis* was shown to contain seven different peptides of three to six amino acids with ACE-I inhibitory activity [72]. In Bulgaria, a prototype of commercial yoghurt containing starter culture of *L. bulgaricus*, *L. helveticus*, and *S. thermophilus* and forming ACE-I inhibitory peptides was developed in 2009 [73]. Then, the study of Dimitrov et al. (2015) revealed the release of Ala-Leu-Pro-Met peptide by *L. helveticus* A1 in white brined cheese, which testifies once again to the valuable properties of this dairy product [67]. It has to be noted that members of the non-starter yoghurt microflora with remarkable proteolytic phenotypes, such as *L. casei* and *L. rhamnosus*, are also capable of releasing ACE-I inhibitory peptides [74].

### 4.3. Antioxidants

Yoghurt and cheese contain antioxidant compounds in varying proportions depending on their processing mode, including lipophilic and hydrophilic antioxidants, such as proteins (casein and its derivatives), conjugated linoleic acid (CLA), microbial enzymes (superoxide dismutase, catalase, and peroxidase), coenzyme Q10, lactoferrin, vitamins (C, E, A and D3), carotenoids, some minerals and some trace elements [75].

The conjugated fatty acids and their composition are subjects of great scientific interest, for instance, conjugated linoleic acid (CLA) is an important nutritional component for weight loss and colon cancer prevention [76]. A recent study by Bosakova-Ardenska (2019) reports that cheese obtained from the milk of the Karakachan sheep breed has an extremely high content of conjugated fatty acids, ranging from 2.12 to 3.53 g per 100 g of fat [77]. Moreover, long-chain fatty acids (Ω-3 and Ω-6) play an extremely important role in the diet of people with coronary and cardiovascular diseases [78]. Dimitrova et al. (2017) reported that the ratio of Ω-6 and Ω-3 fatty acids in white brined cheese from goat’s milk is between 2.91 and 4.09 g per 100 g of fat. Since the optimal value is below 5.0, this dairy product meets the recent requirements of the modern concept of rational nutrition [79]. Ivanova et al. (2015) studied the change in biologically active substances in white brined cheese produced in the Western Rhodopes for two years. They found that the content of vaccenic acid (an essential fatty acid) remained unchanged and with health-promoting values. The concentration of CLA in the first year ranged between 2.67 and 3.32 g per 100 g of fat, while during the second year it varied around 2.40 g per 100 g of fat [17].

A recent metabolomic study of the TwinsUK cohort revealed four metabolites accumulated in volunteers’ blood after the intensive consumption of dairy products. These are diacylphosphatidylcholine and sphingomyelin (containing ceramide), whose presence is associated with butter and sour cream eating; as well as uridine, and trimethyl-N-aminovalerate detected after prolonged low-fat milk intake [80]. The first two are beneficial metabolites that have always been used in the treatment of ulcerative colitis [81] and multiple sclerosis [82].

Trimethyl-N-aminovalerate is a product of 5-aminovalerate deriving from lysine and proline degradation by the gut microflora [83]. It is structurally similar to carnitine, a molecular shuttle taking part in energy generation using long-chain fatty acids.

Uridine is an important metabolite in human breast milk and is abundant in bovine dairy products in the form of uridine-5’-monophosphate (5′UMP), reaching 90% of all nucleotides in the product. Higher blood levels of uridine were found to improve the condition of the arteries [84]. In addition, circulating uridine modulates the phosphorylation of endothelial nitric oxide synthase, which is the main protective enzyme against hypertension, hypercholesterolemia, and diabetes mellitus caused by the reactive oxygen species in the vascular wall [85].

Wall fractions of *L. helveticus* were purified and showed strong scavenging activities on three kinds of radicals and chelating activities on ferrous ions [86]. Another powerful scavenger of hydroxyl radical found in Bulgarian yoghurt is indole-3-propionic acid (IPA), a neuroprotector applied in a novel therapy for Alzheimer’s disease. IPA is produced in high amounts by LAB of the accompanying microflora, especially by *L. helveticus, Leuc. mesenteroides,* and *P. acidilactici* [41].

### 4.4. Exopolysaccharides (EPS)

Bulgarian yoghurt and white brined cheese are rich sources of strains producing exopolysaccharides (EPS), macromolecular compounds known for their anticancer and immunomodulatory activity, as well as the ability to maintain the intestinal barrier by regulating the functions of the gut microbiome [87,88].

Recent screenings evaluated *L. delbrueckii* ssp. *bulgaricus* (154 mg/L EPS), *L. helveticus, L. fermentum, L. rhamnosus,* and *S. thermophilus* (50 to 350 mg/L EPS) as good EPS producers [10,89]. In *S. thermophilus*, EPS are composed of galactose and glucose, while the EPS produced by *L. delbrueckii* ssp. *bulgaricus* contain glucose, galactose, and rhamnose. EPS could be neutral and acidic [90,91]. 

Makino et al. (2010) observed the immunostimulatory activity of acidic high molecular EPS (H-APS) of *L. bulgaricus* OLL1073R-1 in mice. EPS were shown to significantly increase the production of interferon-γ in splenocytes [92]. Oral administration of H-APS to mice led to the augmented activity of the natural killer cells. The same effect was repeated with the oral administration of yoghurt fermented with a starter combination of *L. bulgaricus* OLL1073R-1 and *S. thermophilus* OLS3059. However, when different strains were used (*L. bulgaricus* OLL1256 and *S. thermophilus* OLS3295), the same effects were not observed, thus suggesting that LAB-derived EPS exhibit strain-specific immunostimulatory action. The same strain, *L. bulgaricus* OLL1073R-1, has also been linked with reducing the risk of catching a common cold in elderly individuals [93].

More recently, the anti-inflammatory effect of *L. delbrueckii* subsp. *bulgaricus* and *S. thermophilus* was demonstrated in vitro. Several strains from both species, isolated from homemade yoghurt produced in non-industrial mountain villages in Bulgaria, were shown to have similar anti-inflammatory profiles (induction of IL-10 and TGF-β, and suppression of IL-8) compared to several species (*L. gasseri*, *E. faecium*, *B. longum*) isolated from the intestinal tract of healthy humans. Thus, yoghurt consumption may have a therapeutic effect on patients with inflammatory bowel disease (IBD) [94].

EPS produced by various other *Lactobacillus* species have also been reported to possess immunomodulatory activity. Two strains, *L. paracasei* subsp. *paracasei*, NTU 101 and NTU 102, have been proposed as mild immune modulators of macrophages [95]. Two EPS fractions produced in skim milk by *L. rhamnosus* KF5 have been identified as potential immunomodulators because they stimulate splenocytes proliferation in vitro [96]. *L. confusus* TISTR 1498 (*W. confusa*) was reported to produce an EPS with a relatively high molecular weight, which was characterized as (1→6)-α-D-glucan, and when hydrolyzed, was able to induce the production of NO and cytokines in RAW264.7 cells [97]. *L. kefiranofaciens* M1, isolated from *kefir*, has been extensively studied and shown to affect the cytokine profile in the murine macrophage cell line and to reduce the allergic airway response in mice [98].

### 4.5. Prebiotics

LAB have a probiotic effect, maintaining the balance and composition of the intestinal microflora. This process is mediated by the LAB’s ability to synthesize and assimilate prebiotics [99,100]. According to the last definition, a prebiotic is a “selectively fermented ingredient that results in specific changes in the composition and/or activity of the gastrointestinal microbiota, thus conferring benefit(s) upon host health” [101]. In dairy products, the most widespread prebiotics are galactooligosaccharides (GOS) and other milk oligosaccharides (MO), whose primary role is to selectively support the development of beneficial microflora within the gastrointestinal system. GOS and MO are contained naturally in the milk of mammals in concentrations of 2–10% (including humans and domestic animals), but could also be commercially produced from lactose by enzymatic reactions [102,103,104]. LAB producing GOS and other health-promoting metabolites observed in traditional Bulgarian dairy products are listed in Table 7. 

GOS are typically composed of two to six galactose moieties linked to molecule lactose by β-glycosidic bonds (Gal(β1–3/4/6)]1−6Gal(β1–4)Glc). They are usually mixed in yoghurt, with some branched structures composed of multiple galactose moieties linked to glucose at the reducing end. MO are even more heterogeneous and are composed of five monosaccharides: D-glucose, D-galactose, *N*-acetylglucosamine, L-fucose, and sialic acid (*N*-acetylneuraminic acid) [105]. A brand-new study by van Leeuwen et al. (2020) ranks goat’s milk products as the richest in MO, ranging from 60 to 350 mg/L, considerably more than those from bovine (30–60 mg/L) and sheep’s milk (20–40 mg/L) [106]. Both GOS and MO stimulate the growth of bifidobacteria, especially of *Bifidobacterium longum* subsp. *infantis*, *B. breve*, *B. longum* subsp. *adolescentis*, *B. bifidum*, and the newly evaluated probiotic species *Akkermansia muciniphila* [104,107,108]. Seven selected strains of *L. delbrueckii* ssp. *bulgaricus* can produce GOS directly in yoghurt, reaching up to 3.05 g/L [10]. GOS are trisaccharides and tetrasaccharides, as the last galactose residues are connected by β(1-4) or β(1-6) glycosidic bonds. The β(1-4) linkage with the lactose core is very unusual but is found in Bulgarian-type yoghurt. However, MO, especially from goat’s dairy products, are more appropriate to enhance the growth of some beneficial strains. The growth of *B. animalis*, *B. longum* subsp. *infantis* ATCC 15697, *L. casei*, and *L. acidophilus* probiotic strains was substantially better in goat’s milk-derived infant formula than in milk supplemented with the established GOS prebiotic [109,110]. 

Other compounds found in dairy products, which have recently attracted attention due to their potential prebiotic properties, are flavonoids and short-chain fatty acids (SCFA) [111,112,113]. The presence of flavonoids may alter the gut microbial composition by growth promotion of specific bacteria, for instance, *A. muciniphila,* which was revealed by developing animal models of obesity [114]. Flavonoids incite the gut microbiota to produce short-chain fatty acids (acetic, propionic, and butyric) in the course of fibres fermentation. SCFA are compounds with conferred metabolic benefits due to their action as signalling molecules and energy sources, influencing the host energy metabolism, glucose-insulin homeostasis, the production of endocrine hormones, and inflammatory pathways [115].

## 5. Conclusions

Traditional Bulgarian dairy products contain unique bacterial microflora that has evolved under specific climatic conditions over the centuries. In addition to the excellent technological qualities leading to wonderful taste, aromas, organoleptic properties and durability, LAB strains, both starter and autochthonous, contribute to the functionality of dairy foods. The beneficial effects of dairy food consumption may be attributed to the biologically active compounds in these products; to the modification of milk components by starter cultures; and, importantly, to the direct consumption of LAB strains as preventive and therapeutic agents. The beneficial effect may be direct, through the interaction of the human body with consumed microorganisms, or indirect, as a result of microbial metabolites generated during the fermentation process. The development of new dairy products should be based on current knowledge of the food-gut axis, as well as new well-thought-out and thorough experiments to reveal the mechanisms by which their metabolites can affect health problems. Research on the probiotic potential of dairy products and the main mechanisms by which LAB contribute health benefits will continue with new findings, both in healthy and disabled people. This approach would turn the food into medicine and the diet into treatment.

## Figures and Tables

**Table 1:** Physical and chemical properties of Bulgarian yoghurt.

Properties	Kind of Milk				
	Cow’s	Sheep’s	Buffalo’s	Goat’s	Mixed
Dry matter (%)					
Full-fat milk	11.8	16.5	16.0	11.0	13.0
Low-fat milk	10.3	-	-	-	-
Milk protein, %	3.2	5.2	4.2	3.0	4.0
Fat (%)					
Full-fat milk	3.6	6.5	7.0	3.0	5.0
Low-fat milk	2.0	-	-	-	-
Total (Thörner) acidity (°T)	90.0–150.0				
Storage temperature (°C)	2.0–6.0				

**Table 2:** Physical and chemical properties of Bulgarian white brined cheese. The data are according to the Bulgarian National Standard BDS 15:2010.

Properties	Type of Bulgarian White Brined Cheese				
	Cow’s	Sheep’s	Buffalo’s	Goat’s	Mixed
Dry matter (%) ^1^	46.0	48.0	48.0	48.0	46.0
Fat in dry matter (%) ^1^	44.0	48.0	48.0	44.0	45.0
Total (Thörner) acidity (°T)					
Cheese	200–270				
Brine	160–180				
Salt (%)					
Cheese	3.5 ± 0.5				
Brine	6–10				
Ripening (%) ^1,2^	14.0	16.0	14.0	14.0	14.0
Energy value (kcal/100 g) ^1^	264	287	287	264	269

^1^ Minimal value; ^2^ The ratio of soluble to total protein.

**Table 3:** Physical and chemical properties of Bulgarian *kashkaval* (BDS 14:2010).

Properties	Kind of milk				
	Cow’s	Sheep’s	Buffalo’s	Goat’s	Mixed
Dry matter (%) ^1^	56.0	58.0	56.0	58.0	57.0
Fat in dry matter (%) ^1^	45.0	50.0	45.0	50.0	46.0
Preservatives	Not present				
Emulsifiers and stabilizers	Not present				
Salt (%)	1.8–3.0				
Ripening (%) ^1,2^	20.0	22.0	20.0	22.0	20.0
Energy value, kcal/100g	335	363	335	363	344
Energy value, kJ/100 g	1402	1519	1402	1519	1439

^1^ Minimal value; ^2^ The ratio of soluble to total protein.

**Table 4:** The change in the main physicochemical parameters of *kashkaval* from cow’s milk after 12 months of storage compared to a sample stored for 1 month at 4 °C. The presented data are according to Markova et al. (2019) and Ivanov et al. (2020) [22,23].

Parameter	Month 1	Month 12 after Storage			
		4.0 °C	1.0 °C	−7.5 °C	−18.0 °C
Moisture content (%)	41.9	41.4	41.3	41.4	41.2
Protein content (%)	21.8	21.9	21.9	22.0	22.2
Fat in dry matter (%)	Without significant change, in the range 34.5–35.1				
NaCl (%)	2.10	2.20	2.10	2.20	2.20
pH	5.55	5.4	5.55	5.55	5.55
Total acidity (°T)	175.0	212.0	178.0	176.0	176.0
WSN/TN (%) ^1^	13.88	29.55	21.28	16.13	14.44
NPN/TN (%) ^1^	9.97	16.02	12.26	10.36	10.08

^1^ WSN/TN, the ratio of water-soluble to total nitrogen; NPN/TN, non-protein to total nitrogen. Both parameters are indicators for the ripening and proteolysis progress.

**Table 5:** Chemical content and viable counts of bacteria and yeasts in *kefir* and *koumiss*.

Parameter	*Kefir*	*Koumiss*
Milk protein	2.7%	~3%
Milk fat	<10%	~2%
Lactic acid	<0.6%	0.7–1.8%
Ethanol	–	0.6–2.5% (*v/w*)
Starter culture (CFU/g)	1 × 10^7^	1 × 10^7^
Yeasts (CFU/g)	1 × 10^4^	1 × 10^4^

**Table 7:** LAB producing health-promoting metabolites observed in traditional Bulgarian dairy products.

Product	Free Amino Acids	Bioactive Peptides	Antioxidants	EPS	Prebiotics	Reference
Yoghurt	*L. bulgaricus* *L. helveticus* *L. fermentum* *L. paracasei* *P. acidilactici*	*L. bulgaricus* *L. helveticus* *L. casei* *L. acidophilus* *S. thermophilus*	*L. helveticus* *Leuc. mesenteroides* *P. acidilactici*	*L. bulgaricus* *L. helveticus* *L. fermentum* *L. rhamnosus* *S. thermophilus* *W. confusa*	*L. bulgaricus*	[10,41,62,72,73,74,75,86,96,97]
Cheese	*L. bulgaricus* *L. helveticus*	*L. rhamnosus* *L. bulgaricus* *Lc. lactis* ssp. *lactis* *Ent. faecalis* *Ent. faecium* *L. plantarum* *L. casei*	*L. helveticus*			[44,69,70]
*Katak*	*L. bulgaricus* *L. helveticus*	*L. brevis*		*L. bulgaricus* *L. helveticus*		[71]
*Kefir*	*L. helveticus*	*L. bulgaricus*		*L. kefiranofaciens*		[66,71]

## Data Availability

Not applicable.
